# Protein Kinase A Activity and Anchoring Are Required for Ovarian Cancer Cell Migration and Invasion

**DOI:** 10.1371/journal.pone.0026552

**Published:** 2011-10-19

**Authors:** Andrew J. McKenzie, Shirley L. Campbell, Alan K. Howe

**Affiliations:** 1 Department of Pharmacology, University of Vermont College of Medicine, Burlington, Vermont, United States of America; 2 The Vermont Cancer Center, University of Vermont College of Medicine, Burlington, Vermont, United States of America; University of Birmingham, United Kingdom

## Abstract

Epithelial ovarian cancer (EOC) is the deadliest of the gynecological malignancies, due in part to its clinically occult metastasis. Therefore, understanding the mechanisms governing EOC dissemination and invasion may provide new targets for antimetastatic therapies or new methods for detection of metastatic disease. The cAMP-dependent protein kinase (PKA) is often dysregulated in EOC. Furthermore, PKA activity and subcellular localization by A-kinase anchoring proteins (AKAPs) are important regulators of cytoskeletal dynamics and cell migration. Thus, we sought to study the role of PKA and AKAP function in both EOC cell migration and invasion. Using the plasma membrane-directed PKA biosensor, pmAKAR3, and an improved migration/invasion assay, we show that PKA is activated at the leading edge of migrating SKOV-3 EOC cells, and that inhibition of PKA activity blocks SKOV-3 cell migration. Furthermore, we show that while the PKA activity within the leading edge of these cells is mediated by anchoring of type-II regulatory PKA subunits (RII), inhibition of anchoring of either RI or RII PKA subunits blocks cell migration. Importantly, we also show – for the first time – that PKA activity is up-regulated at the leading edge of SKOV-3 cells during invasion of a three-dimensional extracellular matrix and, as seen for migration, inhibition of either PKA activity or AKAP-mediated PKA anchoring blocks matrix invasion. These data are the first to demonstrate that the invasion of extracellular matrix by cancer cells elicits activation of PKA within the invasive leading edge and that both PKA activity and anchoring are required for matrix invasion. These observations suggest a role for PKA and AKAP activity in EOC metastasis.

## Introduction

Epithelial ovarian cancer (EOC) is the fifth most common cause of cancer deaths in women in the United States and has the highest mortality rate of all gynecologic cancers [1]. Approximately 80% of patients present with late stage disease and less than 25% of those women are cured. EOC constitutes the overwhelming majority (>90%) of ovarian malignancies and is unique in its clinically occult dissemination and metastasis [2]. In contrast to most other types of carcinoma, dissemination of EOC through the vasculature and formation of truly distal metastases is a rare occurrence. EOC cells shed from the primary tumor on the surface of the ovary and exfoliate into the peritoneal cavity. The anatomic placement of the ovaries facilitates local spread of EOC, which can occur by direct migration and invasion of tumor cells to and into adjacent organs, as well as through transport of exfoliated tumor cells throughout the peritoneal cavity by normal peritoneal fluid flow [2], [3]. Because of this unique and furtive mode of dissemination, efforts in developing methods for early detection of EOC have been largely unsuccessful [4], [5], [6]. Despite cytoreductive surgery, combination chemotherapies and strategies for determining serum biomarkers, local and disseminated chemoresistant cells persist and eventually flourish, leading to the high recurrence and <25% five-year survival rate of patients with EOC [3], [5], [7], [8], [9]. Thus, a better understanding of the cellular and molecular mechanisms governing EOC dissemination and invasion has the potential to have significant impact on the course of the disease.

Due to the dependence of EOC dissemination and metastasis on cellular migration and invasion, our laboratory has endeavored to understand the mechanisms underlying these processes in EOC. Cell migration is a highly ordered process that requires coordinated effort between numerous proteins in distinct subcellular regions [10], [11], [12]. We have previously shown that the cAMP-dependent protein kinase (PKA) is important for cell migration and leading edge dynamics in a number of cells [13], [14], [15]. PKA is a heterotetrameric kinase that can exist in two isoforms, type-I and type-II, depending on the isoform of regulatory (RI and RII) subunit associated with the holoenzyme. PKA has numerous targets associated with actomyosin based cell movement [16] and early studies showed that hyper-activation or inhibition of PKA inhibited chemotactic cell migration [16], [17], [18]. This seemingly contradictory role for PKA was clarified by the demonstration that, in addition to overall activity, localization of PKA in subcellular space, mediated through the binding of PKA R subunits to A kinase anchoring proteins (AKAPs; [19], [20]), regulates cell migration [13], [21], [22]. Specifically, it has been shown that PKA subunits and activity are both enriched in protrusive structures at the leading edge of migrating cells [13], [21], [22]. Moreover, broad inhibition of type-I and type-II anchoring (*i.e.* anchoring through RI or RII subunits) in general [13], [21], or specific inhibition of individual anchoring proteins (*e.g.* AKAP-Lbc; [22]), inhibits migration. These and other reports (reviewed in [23], [24]), establish the importance of localizing PKA activity to distinct subcellular locations during normal cell migratory processes.

Underscoring the potential importance of PKA in ovarian cancer pathogenesis are the observations that PKA activity and subunit expression are often dysregulated in EOC. Expression of the PKA catalytic subunit [25] and regulatory RI subunit [26], [27], [28] correlates with advanced stage and/or more aggressive EOC. Moreover, the mRNA levels of AKAP3 (*aka* AKAP110 or fibrous sheath protein of 90 kDa (FSP90)) in ovarian tumors positively correlate with disease stage and poor prognosis [29], [30], while two other AKAPs – AKAP1 (*aka* AKAP149) and AKAP13 (*aka* AKAP-Lbc) – also appear to be up-regulated in mucinous EOC (A. Howe, unpublished observations from Oncomine). Despite these observations, the functional significance PKA and AKAP signaling in metastatic EOC cells has not been well investigated.

Given the importance of PKA and AKAP function for cell migration and their dysregulation in EOC, we investigated the spatial regulation of PKA activity in migrating EOC cells and determined whether PKA activity and anchoring are required for EOC cell migration. Here, we report that PKA activity is up-regulated in the leading edge of EOC cells not only during migration but also during extracellular matrix invasion, as well. Further, inhibition of PKA activity and AKAP function blocks EOC cell migration and invasion. These observations demonstrate, for the first time, the importance of PKA anchoring for matrix invasion and establish the importance of spatially regulated PKA activity in the migration and invasion – and thus perhaps metastasis – of EOC cells.

## Materials and Methods

### Reagents and Cell Culture

SKOV-3 and COS-7 cells were obtained from American Type Culture Collection and maintained in antibiotic-free Dulbecco's modified Eagle's medium (DMEM) supplemented with 10% (vol/vol) fetal bovine serum (FBS). HIO-80 cells were a generous gift from Dr. Andrew Godwin (Department of Molecular Oncology, Fox Chase Cancer Center) and were maintained in a 1∶1 mixture of antibiotic-free Medium 199:MCDB-105 supplemented with 4% FBS and 0.2 U/ml porcine insulin. All cells were grown at 37°C in a humidified incubator containing 5% CO_2_. DMSO, cytochalasin D and trypan blue were purchased from Sigma-Aldrich (St. Louis, MO). GM6001 was obtained from Millipore (Billercia, MA). Phenol red-free Matrigel and human fibronectin were acquired from BD Biosciences (Bedford, MA). Pharmacological inhibition of PKA activity was achieved using H89 or mPKI (Biomol). H89 is an isoquinolone that competes with ATP for binding to PKA; despite widespread use and considerable potency, it exhibits only modest specificity for PKA [31]. mPKI, a highly-specific PKA inhibitor, is a myristoylated, cell-permeable peptide comprising the inhibitory pseudo-substrate region (amino acids 14-27) of the endogenous Protein Kinase A Inhibitor protein (PKI) [14]. Pharmacological inhibition of PKA anchoring was achieved using StHt31 (Promega), a stearated peptide comprising the PKA R-subunit binding domain from Ht31 (*aka* AKAP-Lbc); thus, StHt31 acts as a cell-permeable competitive inhibitor of the interaction between endogenous AKAPs and both RII and, at somewhat higher concentrations, RI PKA subunits [32], [33]. Genetic inhibitors of PKA activity and of type-I or type-II PKA anchoring are described below.

### Plasmids and Transfection

The plasmid encoding mCherry-fused to the PKA inhibitor protein PKI (see above) has been described previously [14], while plasmids encoding GFP fused to the type-I or type-II anchoring inhibitors (RI anchoring disrupter (RIAD; [34]) and superAKAP*-in silico* (sAKAP*is*; [32], respectively), as well as their corresponding scrambled (*scr*) negative controls, were generated using the 5′ phosphorylated oligonucleotides (Invitrogen) listed in Table S1. Specifically, complementary oligos were annealed, generating 5′ *Sal*I and 3′ *BamH*I sticky ends, then ligated directly into *Sal*I/*BamH*I-digested pEGFP-C1 (Clontech). mCherry versions of RIAD and sAKAP*is* were generated by replacing the *Nhe*I-*BamH*I fragment encoding EGFP in the respective plasmids with the *Nhe*I-*BamH*I fragment from pmCherry-C1. For transfection and transient expression of proteins, cells were transfected using Fugene6 (Roche) according to the manufacturer's protocol. Briefly, 6 µl of Fugene6 was added to 100 µl serum and antibiotic free DMEM, briefly vortexed, and incubated at room temperature for 5 min. A total of 1.5 µg DNA was added to the mixture, briefly vortexed, and incubated at room temperature for 15 min. The mixture was then added drop-wise to a 35 mm dish containing cells at between 80-90% confluence, allowed to stand at RT for 2-3 min, then returned to the incubator. All experiments were done between 24-48 h after transfection.

### Immunofluorescence

For visualization of actin, fibronectin, paxillin, and PKA RIα and RIIα subunits, cells were fixed in 3.7% formaldehyde in PBS for 10 min, permeabilized for 10 min in PBS containing 0.25% triton ×100, blocked with PBS containing 3% BSA either for 1 h at RT or overnight at 4°C then incubated with anti-fibronectin (1∶400, BD Transduction), anti-paxillin (1∶500, BD Transduction), anti-PKA RIα or RIIα (Genetex 1∶200 and BD Transduction 1∶200 respectively) primary antibodies for 1 h at RT. After washing 3×5 min with PBS, cells were incubated with Alexa-594 coupled donkey anti-mouse secondary antibody (Invitrogen, 1∶400) and Alexa-488 conjugated phalloidin (Invitrogen, 1∶100) for 1 h at RT. Coverslips were mounted onto glass microscope slides (Fisher) using a small volume of PermaFluor mountant (Thermo Scientific). Epifluorescence images were captured though 10, 20, 40, and 60X Plan Apo objectives on a Nikon Eclipse TE-2000E inverted microscope using the appropriate fluorophore-specific filters (Chroma Technology Corp, Rockingham, VT) and a CoolSnap HQ camera (Photometrics, Tucson, AZ) controlled by Elements (Nikon) software.

### Wound-healing migration assay

Glass coverslips were sterilized by consecutive soaking in 30 min intervals in increasing concentrations (70, 95, 100%) of EtOH. The coverslips were removed from EtOH and air-dried in the tissue culture hood. Once dried, the sterile coverslips were coated in 20 µg/ml human fibronectin diluted in sterile PBS either overnight at 4°C or 2 h at 37°C. A confluent monolayer of cells was seeded onto ECM conjugated coverslips and allowed to adhere overnight. A wound was made in the monolayer by scraping a sterile p200 pipette tip across the cells, and the coverslips were washed once with sterile PBS to remove any cell debris. Images were acquired using a 10x Plan Apo objective on the Nikon Eclipse TE-2000E inverted microscope as described above.

### Trypan Blue Exclusion Assay

To assess cell viability, a 1∶1 mixture of trypan blue (0.4%, Sigma) and serum free medium containing 1% BSA was added to the cells and incubated at room temperature for 10 min immediately after either removing the donut or creating the wound in the monolayer. Images were taken of the periphery of the donut and along the entire margin of the wound using a 10X Plan Apo objective on the Nikon Eclipse TE-2000E as previously described.

### Fabrication of silicone gaskets

Forty g of Sylgard 184 silicone elastomer (Ellsworth Adhesives) and 4 g of Sylgard 184 elastomer curing agent were rigorously mixed until cloudy with bubbles. The cloudy mix was poured into a sterile 10 cm tissue culture dish to a depth of 2-4 mm in height. Bubbles were removed by placing the dish in an 850 ml Desi-Vac container (Fisher) and evacuating the chamber 60 times and allowing the chamber to stand for 10 min. This step was repeated twice or until the mixture was free of bubbles. The silicone mixture was allowed to cure at room temperature for 48 hrs or for an hour on an 84°C hot plate until completely polymerized. The silicone template was gently removed from the dish and placed on a clean dry surface suitable for cutting. To fabricate the donut-shaped gasket, a 6 mm biopsy punch was used to generate a silicone cylinder, and a 2 mm biopsy punch was used to make a smaller hole in the center of the 6 mm cylinder. The silicone “donuts” were then washed in Liquinox (diluted 1∶10 in nanopure water) for 15 min, rinsed 10 times with nanopure water, washed with 70% EtOH for 15 min, then stored in fresh 70% EtOH until needed.

### Donut Migration/Invasion Assay

Sterile glass coverslips were coated with 20 µg/ml fibronectin as described above. Clean, sterile silicone donuts were allowed to air dry before placing onto the coated coverslips. A change in refraction at the donut-coverslip interface can be seen as the donut adheres by conformal contact. Subconfluent cells were serum-starved the night before the assay. The cells were trypsinized and resuspended in the appropriate serum free medium containing 1% BSA and counted using a hemocytometer. Cells were plated at confluence (6000 cells/donut for SKOV-3, COS-7, and HIO-80) in a 10 µl volume using a sterile gel-loading tip into the center of the donut-shaped gasket so as to contain them within a confined area. The number of cells required to form a confluent monolayer depends on the ECM chosen, the diameter of the inner well of the donut, and the spreading ability of the cells, and thus needs to be determined empirically for each cell line. Sterile PBS was added around the gasket to prevent evaporation of the small volume of media. After adhering overnight, the PBS was aspirated, and the donuts were carefully removed using sterile forceps. The nuclei were stained using Hoescht 33342 nuclear stain (Invitrogen, 1∶2000 in media) for 15 min at 37°C. The staining media was removed and replaced with either complete media or media containing pharmacologic agents as described in the figure legends. For invasion assays, the Hoescht-stained monolayer was overlaid with phenol-red free Matrigel and incubated for 15 min at 37°C to allow polymerization. The embedded cells were then supplemented with complete or treated media as in the migration assay. Initial images were acquired through a 2X PlanApo objective on a Nikon Eclipse TE-2000E inverted microscope as described above. Cells were either treated with 10 ng/ml EGF or high serum (10%) to stimulate migration. Additional images were acquired after the indicated times and initial and final state image pairs were analyzed using the default settings of a custom written ImageJ macro (DonutQuant available as Text S1). Briefly, the macro centers and thresholds both initial and final state images then removes far-outlier cells, *i.e.* cells outside the circular monolayers that are too far to be attributable to migration (*e.g.* cells that have loosened and floated in from another area of the dish). A mask of the centered, initial image is then created and superimposed on the final state image and all of the nuclei outside the border of the initial image mask are counted as migrated cells.

### FRET Imaging

SKOV-3 cells expressing the PKA activity biosensor, pmAKAR3 [35], [36] were rinsed and maintained in Leibovitz L-15 medium for FRET imaging. Cells were imaged on a Nikon Eclipse TE-2000E microscope with a 60×/1.4 NA Plan Apo oil-immersion objective lens and cooled CCD camera. CFP, YFP and FRET images were acquired 10 hrs after the start of the assay. The acquisition was set with 700 ms exposure and 2×2 binning for all three acquisitions. Images in each channel were subjected to background subtraction, and ratios of yellow-to-cyan color were calculated using the FRET Analyzer ImageJ plugin. Pseudocolor images were generated using the ImageJ ‘Ratio’ look up table. Images were background-subtracted using the “Subtract background” feature in ImageJ, with a rolling ball radius of 500.

## Results

### PKA activity is increased at the leading edge of randomly migrating SKOV-3 cells

**Figure 1:**
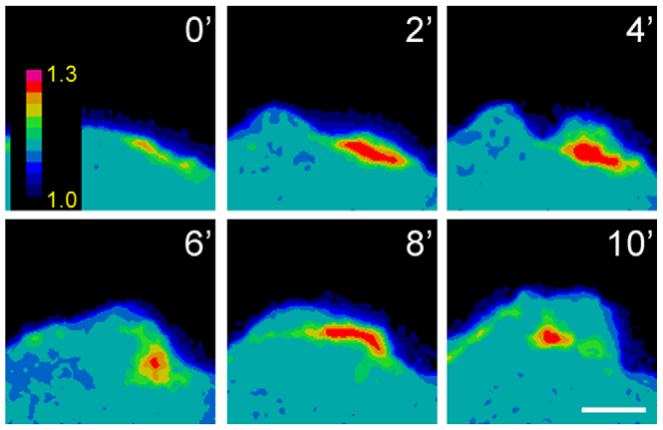
PKA is activated at the leading edge of migrating SKOV3 cells. SKOV-3 cells were transfected with pmAKAR3, plated onto fibronectin-coated dishes overnight, stimulated with 10 ng/ml EGF for 4-6 h, and then imaged by FRET microscopy. Images were captured every 30 sec, then pseudocolored according to FRET ratio (color scale shown in frame 0′): this montage depicts frames 2 min apart. For the whole sequence, see Video S1. Scale bar  = 5 µm.

Previous work had demonstrated discrete activation of PKA in the migratory leading edge of many [13], [21], [22] but not all [37] cell types. To determine if PKA was activated at the leading edge of migrating EOC cells, SKOV-3 human EOC cells were transfected with pmAKAR3, a plasma membrane-directed FRET biosensor for PKA activity [21], [36], then plated on FN-coated glass coverslips, stimulated with 10 ng/ml EGF for 4-6 hrs (to promote random, chemokinetic migration), and monitored by live-cell imaging. PKA activity (as indicated by elevated FRET ratios) was, indeed, discretely and dynamically elevated within the leading edge of migrating SKOV-3 cells (Figure 1 and Movie S1). This suggested that local increases in PKA activity play a role in regulating leading edge dynamics, and thus the migration of actively migrating EOC cells. This hypothesis was supported by early, exploratory experiments in which SKOV-3 cells were subject to wound-healing or scratch migration assays in the absence or presence of widely used inhibitors of PKA activity (H89 and mPKI; see Materials and Methods)) and anchoring (StHt31; see Materials and Methods). Examination of cell morphology shortly after the start of these assays showed significant effects of these inhibitors on leading edge morphology and migration (Figure S1). Specifically, untreated cells migrated into wounded areas efficiently and with broad, ruffling lamellipodia (Figure S1A and D). In contrast, cells in which PKA activity was inhibited with the non-selective PKA inhibitor H89 or the highly-selective inhibitor mPKI did not migrate efficiently and barely entered the wound space, exhibiting leading edge structures devoid of ruffles but rife with focal adhesions – evoking an adhesive, spreading phenotype more than one of migration (Figure S1B and D). Cells in which PKA anchoring was disrupted by StHt31 entered the wound space to a greater extent than H89- or mPKI-treated cells but, unlike control cells, exhibited multiple, erratically-spaced ruffling edges per cell (Figure S1C and D). Together, these results further supported the hypothesis that EOC cell migration might require PKA activity and anchoring and warranted further investigation using more sophisticated approaches and specific reagents.

### EOC cell migration is regulated by activity and anchoring of PKA

**Figure 2:**
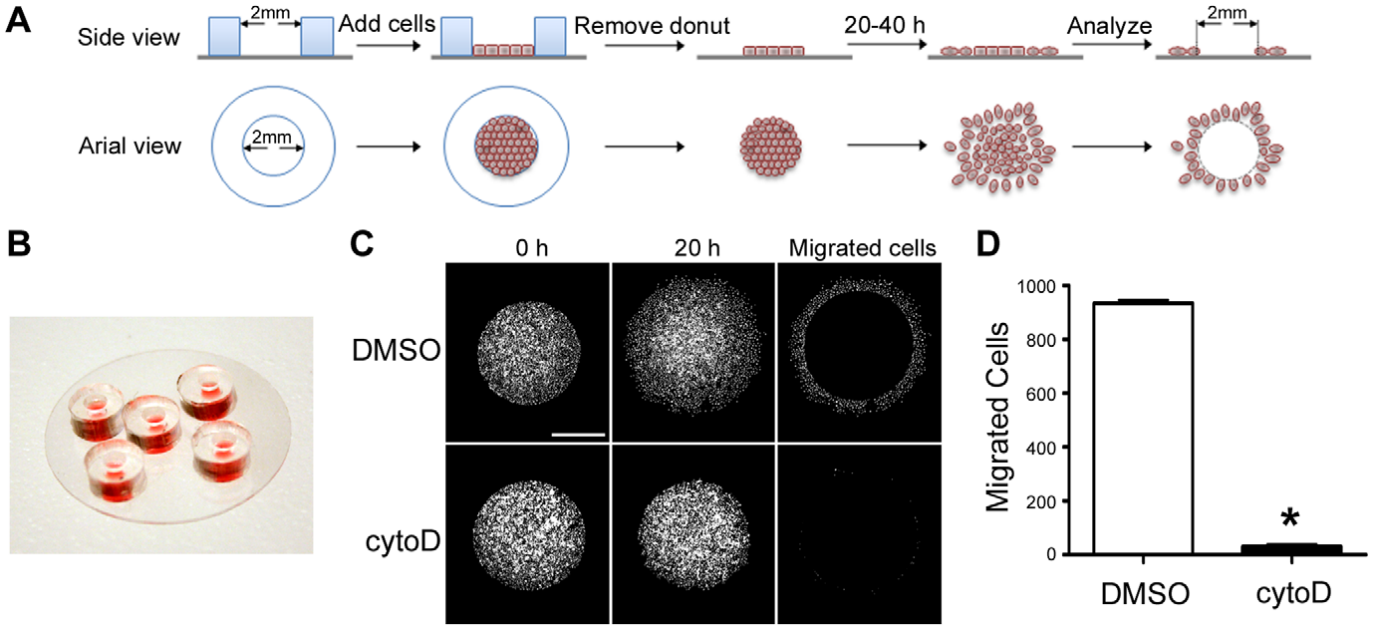
A modified radial migration assay – the Donut assay. (**A**) A schematic representation of the donut migration assay in which cells are seeded at confluence on an extracellular matrix-coated glass coverslip within a donut-shaped silicone gasket. After the cells have stably adhered, the gasket is removed, allowing the cells to migrate radially. Images of the monolayer are captured immediately after donut removal and at any time point thereafter that is suitable to allow migration of a given cell line. A custom ImageJ macro uses the initial image as a subtractive mask to determine the number of migrated cells in the final image taken at the end of the assay. Details are given in Materials and Methods. (**B**) A picture of five donut gaskets on a single 25 mm coverslip; this allows facile setup of replicate assays to increase throughput and generate statistically significant data. (**C**) COS-7 cells were subject to the donut migration assay in the presence of 2 µM cytochalasin D (cytoD) or DMSO as a vehicle control. Representative images taken at the beginning (0 h) and end (20 h) of the assay, as well as masked (final minus initial) images depicting migrated cells are shown. (**D**) The number of migrated COS-7 cells was calculated using the ImageJ macro as described in Materials and Methods. Data represent the mean ± S.E. of five donuts per coverslip with three separate coverslips per treatment. (* = P<0.001).

Wound-healing migration assays, while inexpensive and easy to implement, suffer from several limitations, not the least of which are fatal injury to cells along the assay front and removal of the underlying extracellular matrix (ECM). Thus, an experimental condition that results in inefficient migration in this assay may reflect, for example, heightened sensitivity to bystander damage or failure to produce *de novo* matrix on which to migrate. Therefore, to further and better explore the requirement of PKA activity and anchoring specifically for EOC cell migration, we developed an improved version of a previously described migration assay [38] that easily and accurately measures the number of cells migrating in a radial manner from a defined circular monolayer (Figure 2; see Materials and Methods for details). Our assay – coined the ‘donut assay’, after the donut-shaped silicone gaskets used to establish the monolayer – is reliable, inexpensive, easy to implement, applicable to nearly any cell type, and scalable to achieve relatively high throughput and statistical significance using a small number of cells (∼6000), relative to other comparable ‘release’ assays (*e.g.* wound-healing assays). In addition, unlike wound-healing assays, our approach significantly reduces both the denuding of the ECM protein covering the migration surface (Figure S2) and the fatal injury to cells on the assay periphery (Figure S3). To increase the utility and ease of implementation of the assay, we developed an ImageJ macro (‘DonutQuant’; see Text S1) to quickly and automatically quantify migration. Briefly, the macro subtracts an initial, ‘time = 0’ image (taken immediately after donut removal) from a later or final image and counts the nuclei outside the ‘time = 0’ area (Figure 2C and D). Experiments using cytochalasin D to inhibit actin dynamics (Figure 2C and D) as well as mitomycin C to inhibit cell cycle progression (data not shown) have confirmed that the assay measures migration and not simply displacement of the monolayer by cell division. We have used this assay to measure migration in a wide variety of cell types (including CHO-K1, COS7, HIO-80, HUVEC, MCF7, MCF10A, MDA-MB-231, MDCK, NIH3T3, REF52, and SKOV-3) with comparable ease and effect (A. McKenzie, S. Campbell, A. Howe, unpublished observations).

Using this assay, we investigated the effects of inhibiting PKA or AKAP function on EOC cell migration by transfecting SKOV-3 cells with plasmids encoding fluorescent proteins (EGFP, mCherry) fused to peptide inhibitors of PKA activity or anchoring (see Materials and Methods). Specifically, we used the fluorescent protein mCherry fused to the PKA inhibitor protein PKI (mCherry-PKI) to inhibit PKA activity [14], and EGFP fused to the RI anchoring disruptor (RIAD) [34] and superAKAP*is* (sAKAP*is*) [32] peptides to specifically disrupt type-I and type-II PKA anchoring, respectively. Plasmids encoding mCherry alone or EGFP fused to scrambled RIAD or sAKAP *is* sequences were used as negative controls. These genetically encoded reagents allow exquisite specificity in the inhibition of PKA activity [14] or inhibition of anchoring through both RI and RII subunits [32], [34] while enabling the detection and monitoring of treated cells through fluorescence microscopy. Distinguishing between type-I and type-II anchoring is appropriate and necessary, as type-I and type-II PKA activity can regulate distinct signaling pathways [19], [20] and, while both types of PKA have been implicated in migration in other systems [13], [21]-[23], [39], neither have been assessed in the context of ovarian cancer migration.

**Figure 3:**
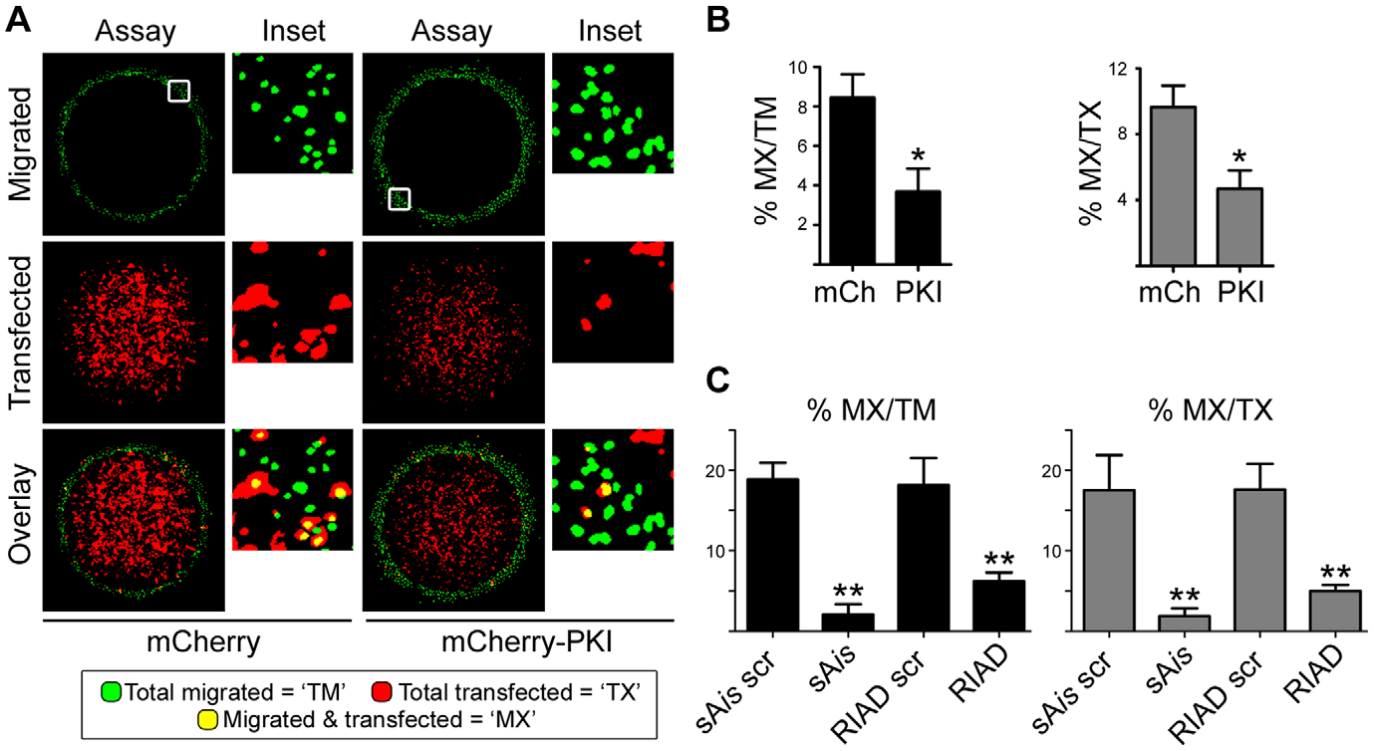
PKA activity and anchoring are required for SKOV-3 cell migration. (**A**) SKOV-3 cells were transfected with either empty mCherry plasmid or mCherry-PKI, then subject to donut migration assays. Representative thresholded images of the migrated cells (Migrated, with nuclei pseudocolored green), the total population of transfected cells (Transfected), and an overlay of these two images are shown. Insets depict enlargements of the areas indicated by the squares in the top panels. Thus, the total migrated cells (‘TM’) are depicted in green, the total transfected cells (‘TX’) are depicted in red, and the yellow nuclei depict transfected cells that migrated, i.e. migrated and transfected cells (‘MX’). (**B**) For cells transfected with either empty mCherry (mCh) or mCherry-PKI (PKI), the average percent (± S.E.) of migrated transfected cells over the total number of migrated cells (‘MX/TM’) was calculated. To normalize for transfection efficiency, average percent (± S.E.) of migrated transfected cells over the total number of transfected cells (‘MX/TX’) was also calculated. (* = P<0.05) (**C**) SKOV-3 cells were transfected with plasmids encoding EGFP fused to inhibitors of type-I (RIAD) or type-II (sAis) PKA anchoring, or their respective scrambled controls (RIAD scr, sAis scr), then subject to donut migration assays and analysis as described in (**A** and **B**). (** = P<0.001)

After transfection, cells were subjected to the donut migration assay and, after 24 hrs, the number of transfected cells that migrated, as well as the total number of transfected cells and total number of migrated (transfected and non-transfected) cells was quantified (Figure 3). Acquisition of these three numbers allows the expression of migration as the percentage of migrated, transfected cells over the total migrated cells (‘MX/TM’) or, to control for differences in transfection efficiency between plasmids, the percentage of migrated, transfected cells over the total number of transfected cells (‘MX/TX’). Using this method, SKOV-3 migration was significantly attenuated when either PKA activity (Figure 3A and B) or anchoring (Figure 3C) was inhibited using the genetic reagents described above. Interestingly, migration was inhibited almost equally by disruption of anchoring through either RI or RII subunits (Figure 3C), consistent with previous reports establishing the importance of both type-I and type-II PKA anchoring in the migration of other cell types [13], [21], [22], [39]. In summary, these data establish the necessity of PKA activity and anchoring for the migration of EOC cells.

### Type-II PKA is responsible for leading edge PKA activity during migration

**Figure 4:**
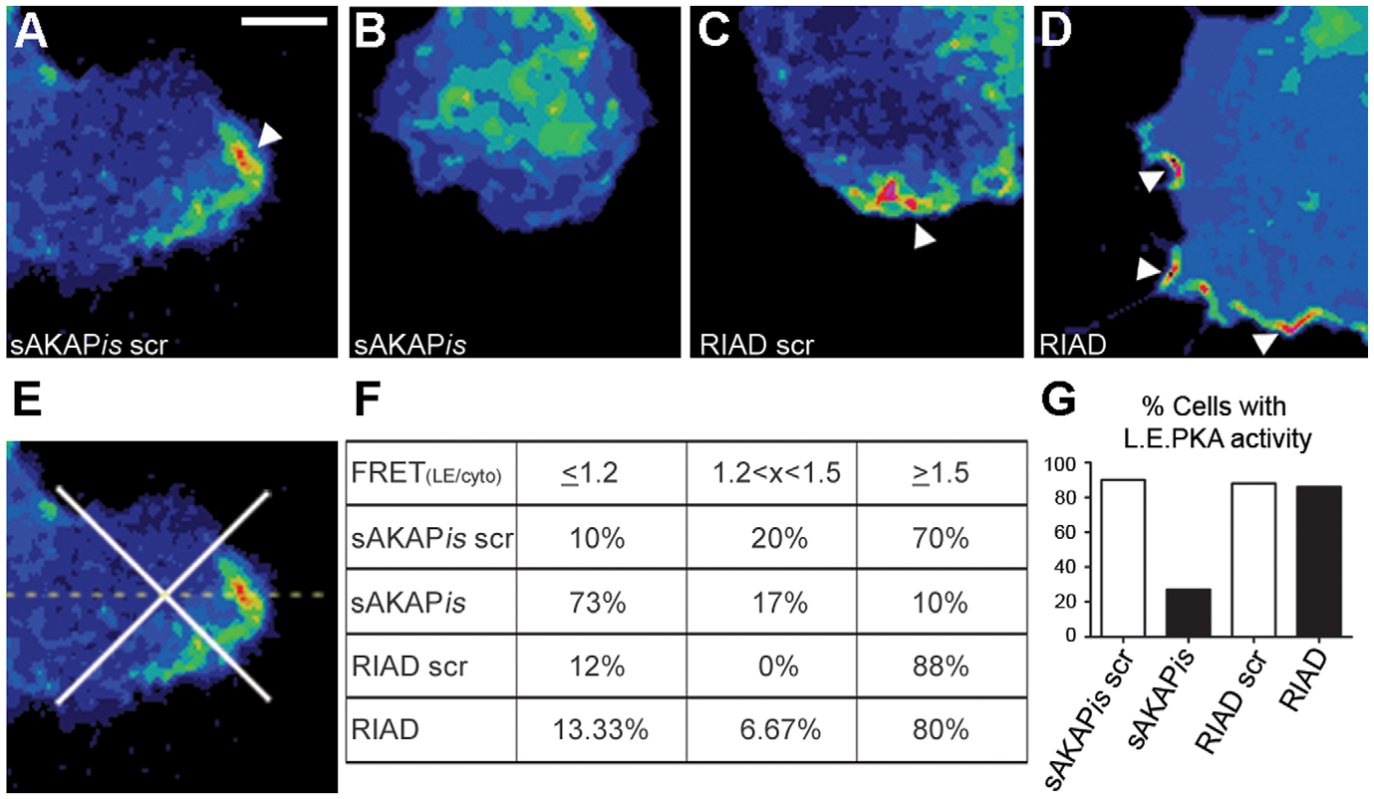
Leading edge PKA activity is mediated by RII-AKAP interactions. (**A-D**) SKOV-3 cells co-expressing the indicated plasmids in conjunction with pmAKAR3 were subject to the donut migration assay for 10 hr and imaged via widefield fluorescent microscopy and FRET microscopy. Representative pseudocolored FRET images of cells co-expressing mCherry fused to (**A**) scrambled superAKAP*is* (sAKAP*is* scr), (**B**) superAKAP*is* (sAKAP*is*), (**C**) scrambled RIAD (RIAD scr), or (**D**) RIAD are shown. White arrow heads point to leading edge PKA activity. Scale bar = 10 µm. (**E**) To quantify the percent of cells exhibiting leading edge PKA activity, the cells were divided into leading edge, cytoplasmic, right and left quadrants, and a ratio of the FRET ratio values was generated *via* linescan analysis through the highest intensity pixels within the leading edge. (**F**) The tabulated results of cells exhibiting leading edge PKA activity is shown as the percentage of cells displaying low (≤1.19), medium (1.2<x<1.5), or high (≥1.5) LE/Cyto FRET ratios. (**G**) shows a graphical representation of the tabulated data where cells are scored as having leading edge PKA activity if the LE/Cyto FRET ratios are >1.2 (n = 15 from three separate experiments).

Given that PKA activity was enriched in the leading edge of migrating SKOV-3 cells and that inhibition of PKA activity and anchoring through both RI and RII stunted migration, we sought to determine whether leading edge PKA activity was mediated through either type-I or type-II anchoring. To assess the effect of RI- or RII-AKAP anchoring disruption on the localization of PKA activity during EOC migration, SKOV-3 cells were co-transfected with pmAKAR3 and plasmids expressing genetic inhibitors of type-I and type-II R-subunit anchoring (RIAD and sAKAP*is*, respectively) fused to mCherry. The mCherry fluorescence spectra does not overlap with those of either YFP or CFP and thus allow these fluorescently-labeled anchoring disruptors to be used in conjunction with FRET microscopy. Once transfected, the cells were subject to the donut migration assay for 10 hrs where upon the cells were imaged via FRET microscopy as described above. Cells expressing both the anchoring disruptor and PKA reporter transfected plasmids were imaged and CFP, YFP, FRET, and mCherry images were acquired. To quantify the percentage of cells exhibiting leading edge PKA activity, the average FRET ratio at the leading edge (FRET_LE_; measured 5-25 µm from the front of the cell) was measured in proportion to the FRET ratio in the cytoplasm (FRET_cyto_; measured 25-50 µm from the front of the cell (Figure 4E)). Ratio values were binned into high (FRET_LE_/FRET_cyto_ ≥1.5), moderate (FRET_LE_/FRET_cyto_ between 1.2 and 1.5), and no (FRET_LE_/FRET_cyto_ <1.2) leading edge PKA activity. Using these strict parameters >80% of cells expressing pmAKAR in addition to mCherry-sAKAP*is* scr, -RIAD, or -RIAD scr showed leading edge PKA activity (Figure 4A, C, D, F and G). However, <30% of the cells co-expressing pmAKAR3 and mCherry-sAKAP*is* showed leading edge PKA activity (Figure 4B, F and G). In these instances, the average FRET ratio was significantly lower than that seen in sAKAP*is* scr controls cells (Figure 4F). Additional observations obtained by immunofluorescent staining of migrating SKOV-3 cells with antibodies against RI and RII PKA subunits revealed a clear enrichment of RII, but not RI, in protrusive leading edge structures (Figure S4), consistent with observations in other cell types [13], [22]. These data, in conjunction with the migration data reported above, demonstrate that PKA is localized to the leading edge of migrating EOC cells and that PKA activity is required for EOC cell migration. Furthermore, though leading edge PKA activity seems to be mediated by RII-AKAP interactions, our data demonstrate a requirement for both RI and RII anchoring in cell migration.

### Type-II PKA mediates leading edge PKA activity at the invading front of SKOV-3 EOC cells

**Figure 5:**
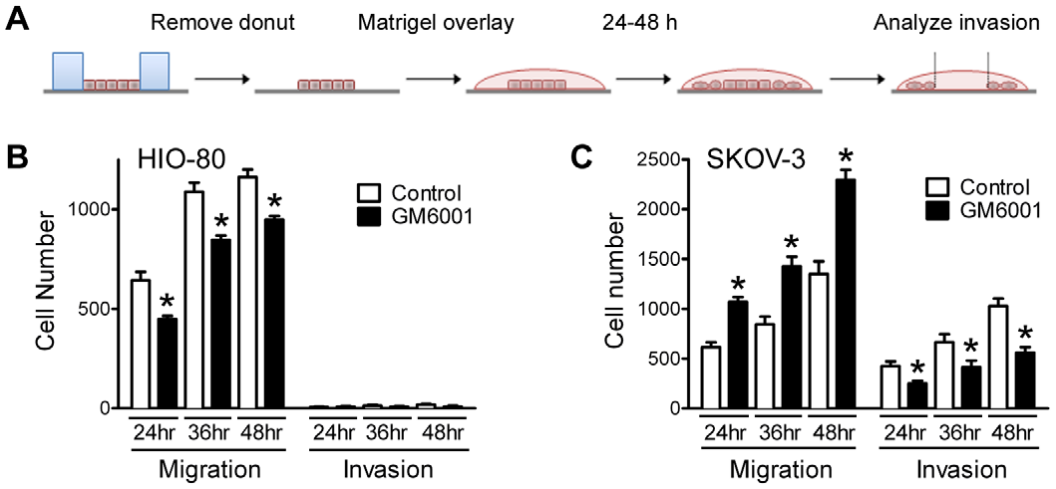
A modification of the donut assay allows measurement of matrix invasion. (**A**) A schematic representation depicting the donut invasion assay. After removal of the silicone gasket, the cell monolayer is covered with a Matrigel overlay, and invasion is analyzed as described for Figure 2. (**B, C**) Migration and Matrigel invasion of non-tumorigenic human immortalized ovarian (HIO-80) epithelial cells (**B**) and tumorigenic SKOV-3 (**C**) was assessed at 24, 36, and 48 h after donut removal, in the presence or absence of the matrix metalloprotease inhibitor GM6001 (25 µM). Graphs represent the mean ± S.E. of the number of migrated or invaded cells. (* = P<0.05)

Late-stage EOC is characterized by disseminated intraperitoneal metastases that form as a consequence of localized invasion of the submesothelial ECM [2], [40]. Invasion through a three-dimensional matrix is a highly ordered process that is unique in both mode and mechanism of action from two-dimensional migration [41], [42], [43], [44]. Importantly, the localization of PKA activity and role of PKA anchoring in cancer cell invasion have never been directly investigated. Thus, to assess whether PKA activity and AKAP function were required for EOC cell invasion, as well as migration, we modified our migration assay to measure invasion by overlaying cell monolayers, immediately after donut removal, with Matrigel - a basement membrane extract commonly used in invasion assays (Figure 5A). While similar approaches have been taken with other chemokinetic migration/invasion assays [45], initial experiments were performed to ensure that this modification assessed true invasion. Specifically, donut migration and invasion assays using the HIO-80 human immortalized ovarian epithelial cell line [46] showed that these non-transformed, non-tumorigenic cells migrate efficiently on a two-dimensional ECM, but are incapable of navigating through the three-dimensional Matrigel overlay (Figure 5B). In contrast, metastatic SKOV-3 cells exhibited readily-detectable migration through the Matrigel overlay (Figure 5C). As expected, the efficiency of movement through the three-dimensional ECM was lower than across a two-dimensional surface, consistent with an increased ‘barrier’ function attributable to the Matrigel. Furthermore, inhibition of matrix metalloproteinases (MMPs) with GM6001 significantly reduced the ability of SKOV-3 cells to invade through the Matrigel (Figure 5C), suggesting that the movement of cells was indeed due to invasion rather than simply migration under the Matrigel. Unexpectedly, while GM6001 inhibited invasion, it also significantly enhanced the migration of SKOV-3, but not HIO-80 cells (Figure 4B and C); we have found no precedent and currently have no explanation for this. Finally, the performance of our assay was comparable to or better than that seen with a traditional modified Boyden chamber/Transwell™ invasion assay; while SKOV-3 cells showed comparable invasive potential, the non-invasive HIO-80 cells also showed low but significant ability to navigate through Matrigel-coated Transwell™ membranes in our hands (data not shown). Taken together, these observations validate this new method as an effective assay to measure tumor cell invasion.

**Figure 6:**
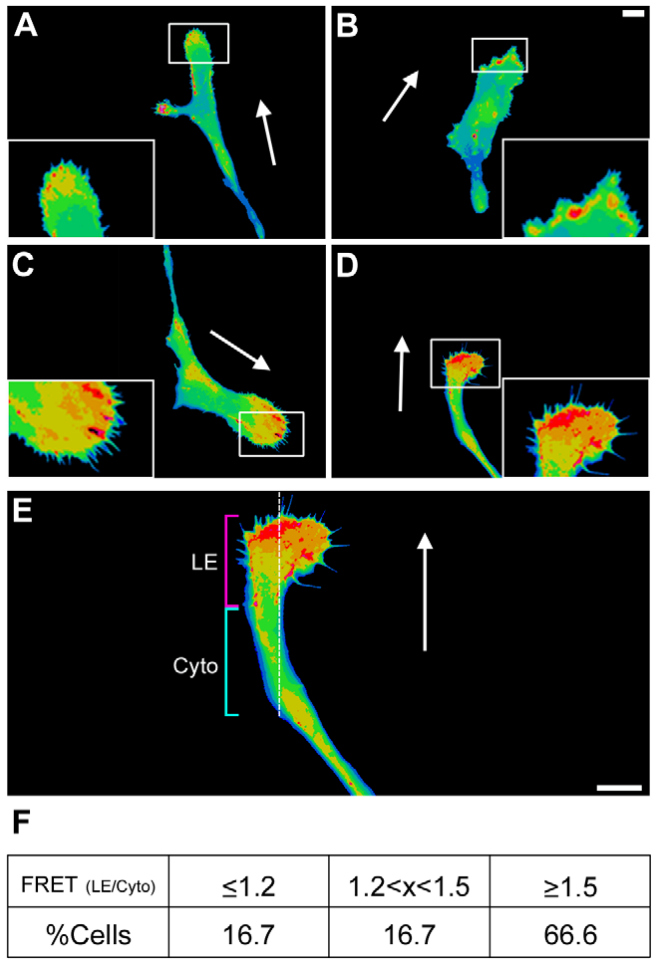
PKA is activated at the leading edge of invading SKOV-3 cells. (**A-D**) SKOV-3 cells transiently expressing pmAKAR3 were subject to the donut invasion assay and images of cells on the monolayer periphery were acquired 10 h after gasket removal. Pseudocolored FRET images are shown, with insets depicting enlargements of the areas indicated by the white rectangles in each panel. White arrows indicate the direction of invasion with respect to the cell monolayer. Scale bar  = 10 µm. (**E, F**) To quantify the number of invading cells exhibiting leading edge PKA activity, the average FRET ratio was measured by linescan in the leading edge (LE) and the cytoplasm (Cyto) of cells and a ratio of these values (FRET(_LE/Cyto_)) was determined. (**F**) shows the percentage of cells exhibiting low, medium, or high LE/Cyto FRET ratios, as described in Figure 4 (n = 28 from four separate experiments).

**Figure 7:**
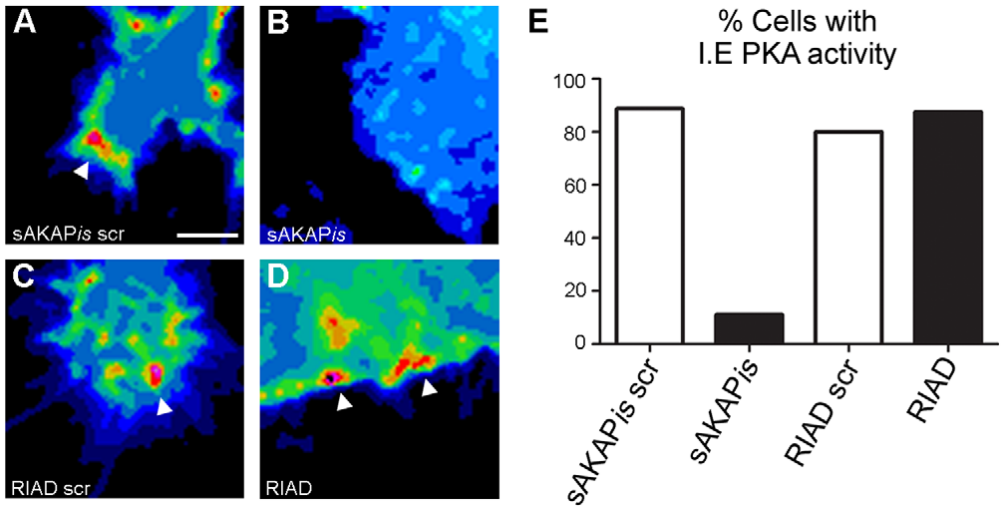
Invading edge PKA activity is mediated by RII-AKAP interactions. (**A-D**) SKOV-3 cells co-expressing the indicated plasmids in conjunction with pmAKAR3 were subject to the donut invasion assay for 18 hr and imaged via FRET microscopy. Representative pseudocolored FRET images of cells co-expressing mCherry fused to (**A**) scrambled superAKAP*is* (sAKAP*is* scr), (**B**) superAKAP*is* (sAKAP*is*), (**C**) scrambled RIAD (RIAD scr), or (**D**) RIAD are shown. White arrowheads point to leading edge PKA activity (scale bar  = 10 µm). (**E**) The results, as the percentages of cells exhibiting PKA activity at the invasive edge (I.E.), were tabulated as in Figure 4 (n = 21 from three separate experiments).

To assess the localization of PKA activity during EOC cell invasion, we transfected SKOV-3 cells with the pmAKAR3 PKA biosensor, subjected them to the donut invasion assay as described above, and acquired FRET images of invasive cells on the assay periphery 18 h after Matrigel overlay (Figure 6). Cursory microscopic analysis of invasive cells revealed elongated morphologies with narrower leading edge structures (compare Figure 6A-D to Figure 1 and Figure S2), often containing multiple filopodia (Figure 6C and D); morphologies consistent with three-dimensional invasion compared to two-dimensional migration. Importantly, the vast majority of invasive cells exhibited either discrete or broad areas of increased PKA activity within their invasive leading edges (Figure 6A-D). To quantify the percentage of cells exhibiting invasive edge PKA activity, the average FRET ratio at the invasive leading edge was measured in proportion to the FRET ratio in the cytoplasm as described for Figure 4. Greater than 80% of the imaged cells displayed an increase in PKA activity in invading edge (Figure 6F). Leading-edge, as well as and global PKA activity was all but eliminated by co-expression of pmAKAR3 with mCherry-PKI, confirming the specificity of the pmAKAR reporter for PKA activity in this assay (Figure S5). Given that invasion often utilizes different mechanisms of motility than does 2D migration, we sought to determine whether the anchoring mechanism driving PKA activity at the invasive leading edge was fundamentally different than that seen for the migrating leading edge. Thus, in parallel to the experiments depicted in Figure 4, SKOV-3 cells expressing pmAKAR3 and fluorescent inhibitors of type-I or type-II PKA anchoring (mCherry-RIAD or mCherry-sAKAPis, respectively) were subject to the donut invasion assay and analyzed by FRET microscopy as described above. The vast majority (∼80%) of cells expressing RIAD or non-inhibitory control peptides for RIAD and sAKAP*is* displayed discrete areas of heightened PKA activity at the invasive leading edge (Figure 7A, C, D and E), while only ∼10% of cells expressing sAKAP*is* displayed significant leading edge PKA activity (Figure 7B and E). These data establish – for the first time – that matrix invasion, like migration, also invokes up-regulation of PKA activity at the leading edge and that this localization is mediated through anchoring of the PKA RII subunit.

### EOC cell invasion requires PKA activity and anchoring

**Figure 8:**
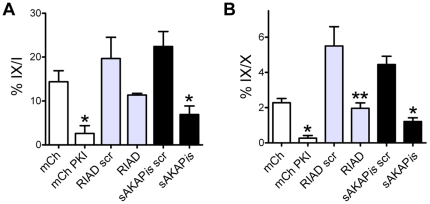
PKA activity and anchoring are required for SKOV-3 cell invasion. (**A, B**) SKOV-3 cells were transiently transfected with plasmids to inhibit PKA activity or anchoring (mCh-PKI, GFP-RIAD, GFP-sAis) or their respective controls (Empty mCh, GFP-RIAD_scr, GFP-sAis_scr), then subject to invasion analysis as described in Figures 3 and 4. (* = P<0.01; ** = P<0.05). In parallel to the method described in Figure 3, invasion was calculated as the average percentage (± S.E.) of invading transfected cells over the total number of invading cells (**A**; ‘IX/I’) or, to normalize for transfection efficiency, the average percentage of invading transfected cells over the total number of transfected cells (**B**; ‘IX/X’).

The previous observations demonstrated that PKA was activated at the leading edge of EOC cells during matrix invasion. To determine whether localized PKA activity was actually required for invasion, SKOV-3 cells were transfected with pmCherry-PKI, pEGFP-sAKAP*is* or pEGFP-RIAD (as described for Figure 3) and subjected to the donut invasion assay (as described in Figure 5) for 24 h. As observed for migration, inhibition of PKA activity and type-II PKA anchoring significantly attenuated SKOV-3 cell invasion (Figure 8A and B, *white & black bars*). Initial analysis of the effect of inhibition of type-I PKA anchoring – by determining the proportion of invaded cells that were transfected (or ‘IX/I’) - showed a trend toward decreased invasion, although this was not statistically different from the scrambled control (Figure 8A, *gray bars*; P = 0.097); however, upon normalizing the data for transfection efficiency (*i.e.* the number of invading, transfected cells over the total number of transfected cells, or ‘IX/X’), there was indeed a statistically significant (P<0.05) inhibition of invasion caused by disruption of RI anchoring (Figure 8B, *gray bars*). Thus, as demonstrated for migration, PKA activity as well as type-I and type-II PKA anchoring are required for the invasion of SKOV-3 cells into a three-dimensional ECM.

## Discussion

Over-expression of various PKA subunits is a hallmark of a vast array of human tumors [47], [48], [49]), including ovarian cancer [26], [27], [28], and often correlates with more severe pathology and poorer prognosis. In this regard, PKA is classically thought to enhance mitogenic signal transduction and promote early cell cycle transition. However, a number of reports have also established a role for PKA in promoting invasion and metastasis in a number of tumor models [50], [51], [52], [53], [54]; however, this line of investigation has not been extended to EOC until now. Our results clearly demonstrate that spatial regulation of PKA activity is a critically important facet of the migration and invasion – and thus, perhaps the metastasis – of EOC cells.

Previous work has demonstrated enrichment of PKA in the leading edge of migrating cells, with the most detailed descriptions being in fibroblasts [13], [21] and breast and colon carcinoma cells [22]. Our data now extends this paradigm to EOC cells and expands the paradigm in an important and substantive way by demonstrating localization of PKA to the leading edge during three-dimensional matrix invasion. Furthermore, we have also demonstrated the requirement for AKAP function for the migration of EOC cells, as has been demonstrated for other cell types [13], [39], [55]. Finally, we have also demonstrated – for the first time - the requirement for PKA anchoring *via* AKAPs for cancer cell invasion of a three-dimensional ECM. A number of previous reports have implicated a variety of AKAPs as positive or negative regulators of invasion and metastasis: these include AKAP9/yotiao [56]; AKAP12/SSeCKS/gravin [57], [58]; AKAP13/AKAP-Lbc [59]; the erzin/radixin/moesin and merlin protein family [60], [61], [62]; and the WAVE (Wiskott-Aldrich syndrome/verpolin-homologous) -1 and 2 proteins [63], [64]. Without exception, however, those reports dealt with gain- or loss-of-function of their respective AKAPs as a whole and did not specifically target only the PKA anchoring domain. This is an important corollary, as the overwhelming majority of known AKAPs – and essentially all of those previously implicated in metastasis – have important functions beyond their ability to anchor PKA [19], [20]. Thus, complete gain- or loss-of-function studies, *i.e.* over-expression or silencing of an entire AKAP, would affect all of its potential functions. In many cases, the functions other than PKA anchoring are quite powerful – *e.g.* guanine nucleotide exchange for Rho family GTPases (AKAP-Lbc/AKAP13); scaffolding other kinases and enzymes (AKAP9, AKAP12); coupling membrane receptors to the cortical actin cytoskeleton (ezrin family proteins); dendritic actin nucleation (WAVE1 and 2) – and could readily contribute to invasion and metastasis in their own right. In the current study, however, we specifically inhibited only the ability of AKAPs to anchor PKA; thus, to our knowledge, ours is the first report to establish a role specifically for PKA anchoring *per se* in events related to metastasis.

An important question arises from the current observations: if leading edge PKA activity is mediated by exclusively by RII-AKAP interactions, as reported here, then why are both type-I and type-II PKA anchoring required for migration and invasion? This question is, at the same time, both reassuring and vexing, as both type-I and II PKA have been previously implicated in cell migration [13], [21], [22], [39], but it is becoming increasingly clear that the two types of PKA have non-overlapping targets and functions [20], [48], [65]. It is also important to note that the current study uses pmAKAR3, a membrane-tethered biosensor for measuring PKA activity, which – by design – does not faithfully report any cytosolic PKA activity that may exist some distance from the membrane. Thus, it is possible that there may be some leading edge PKA activity, within the thin layer of cytoplasm that is distant from the membrane, that is attributable to RI-AKAP interactions. Perhaps more likely is that, during migration, there is significant PKA activity within the cytosol that is both not detected by the membrane-tethered pmAKAR3 and not as ‘concentrated’ (in terms of specific PKA activity per total cellular protein) as the activity within the leading edge [13], [21], [22], [39]]. Thus, while RII-AKAP complexes preferentially regulate events within the leading edge, RI-AKAP complexes may preside over myriad events within the rest of the cell – outside the leading edge – that also ultimately contribute to migration/invasion. Delineating the precise deficits in migration and invasion associated with type-I and type-II PKA would best require a complete catalog of the specific molecular targets for each type of PKA activity and an understanding of what role each target plays in migration/invasion; at present, this is quite unfeasible. More reasonably, however, one could begin to empirically address the matter through more frequent imaging and closer correlation of cell morphology and PKA activity over the entire duration of migration and invasion, and subsequent observation of discrete changes in these parameters in the absence of type-I or type-II anchoring.

While we have clearly demonstrated that PKA and AKAP function are both required for EOC cell migration and invasion, the discrete molecular targets for PKA as well as the specific AKAP(s) responsible for PKA localization in these cells are unknown. Delineation of specific signaling pathways that link PKA to migration and invasion is made difficult by the considerable and growing number of cytoskeleton- and/or migration-associated PKA substrates [16], [23] and anchoring proteins [19], [20], [24]. It is further complicated by the fact that, as discussed above, inhibition of either type-I or type-II PKA anchoring blocks migration and invasion, likely through different effectors. Furthermore, while not the focus of the current study, it should be noted that, as a kinase regulated by intracellular cAMP, PKA is quite likely to work in concert with the other major cAMP effectors, the Epacs (*e*xchange *p*roteins *a*ctivated by *c*AMP), which can form multi-component scaffolds with PKA to mediate signaling events in response to cAMP [66], [67] and also have considerable influence over cell adhesion, migration and related events [68], [69]. This possibility notwithstanding, the current study unequivocally establishes a role for PKA itself, in a paradigm involving its localization through AKAPs, in the regulation of ovarian cancer migration and matrix invasion.

While our data demonstrate the necessity for PKA activity and anchoring during migration as well as invasion, it is not known whether the targets for PKA during migration will be the same as those in invasion. Although the processes are intimately related, migration can occur without invasion, but invasion cannot occur without migration, and the exact mechanism of migration during invasion may differ as a function of cellular phenotype and microenvironmental cues [64]-[67]. Given our growing appreciation of the differences between cell migration in two-dimensions vs three-dimensions [41], [42] and the differences between normal and tumor cell migration [43], [44], it would not be surprising if distinct (but, likely, somewhat overlapping) sets of PKA substrates and anchoring proteins were called upon to specifically regulate the processes involved in EOC cell invasion compared to migration. An intriguing example would be a requirement for localized PKA in regulating the expression, secretion or function of ‘invasion-specific’ proteins such as matrix metalloproteases [52]. Nonetheless, the identification of the substrates and anchoring proteins for PKA that are relevant to migration and invasion are an ongoing focus for several laboratories, including our own. Indeed, propitious initial candidates for relevant AKAPs would be those that appear to be up-regulated in various forms of EOC AKAP3/AKAP110 [29], [30]; AKAP1/AKAP149 and AKAP13/AKAP-Lbc (A.K. Howe, unpublished observations)).

Although it only accounts for approximately 3% of all cancer diagnoses in the United States, ovarian cancer is the 5^th^ leading cause of death among women [1]. This is largely due to the fact that most ovarian cancer patients become symptomatic - and are therefore diagnosed - after the tumor has metastasized. The metastatic spread of EOC, like other cancers, is completely dependent on the acquired ability of tumor cells to undergo invasive cell migration [2]. In this work, we have shown that both migrating and invading EOC cells exhibit enriched PKA within their leading edge and that both PKA activity and its localization through AKAPs are required for migration and invasion of EOC cells. This is the first demonstration of an association of localized PKA activity and the process of matrix invasion. More importantly, these observations add important new molecular insight into some of the most clinically relevant cellular behaviors associated with the pathogenesis of EOC. To be certain, the *in vitro* mode of matrix invasion assessed in the current study does not recapitulate the full course and associated mechanisms of EOC metastasis *in vivo*. Especially relevant in this regard are the complex biology of both the interaction of metastatic EOC cells with their target microenvironment, which consists of mesothelial cells and fibroblasts in addition to ECM components [40], [70], and of multicellular tumor spheroids, regarded as the minimal metastatic unit of EOC [71]. Nonetheless, the current studies provide a solid foundation that justifies the further exploration of the role of PKA and AKAP function in higher-order models of EOC metastasis.

## Supporting Information

Figure S1 **PKA activity and anchoring are required for normal leading edge morphology in migrating SKOV-3 cells.** (**A-C**) Confluent monolayers of SKOV-3 cells plated on FN-coated coverslips were wounded by scratching and re-fed with media containing DMSO (**A**, Ctrl), 10 µM H89 (**B**), or 50 µM StHt31 (**C**). Cells were allowed to migrate into the wound for 4 h before fixation and staining to visualize F-actin (green) and the focal adhesion proteins VASP (red) and vinculin (blue). (**D**) SKOV-3 cell monolayers were cultured and wounded as described above, then treated with DMSO (Ctrl), H89, StHt31, or 25 µM mPKI. After 4 h, the cells were fixed and stained as above and the number of leading edge lamellipodia in cells at the wound edge was quantified. Values represent the means ± S.E. from at least three experiments (n>100; * = P<0.001).
(TIF)

Figure S2 **The donut migration assay preserves ECM protein coating better than scratch assays.** (**A**) COS-7 cells were plated on coverslips coated with 20 µg/ml FN and subject to either the donut migration assay or a scratch migration assay for 2 hrs. Coverslips were fixed and processed for immunofluorescence using an antibody against FN, fluorescent phalloidin to stain F-actin, and DAPI to stain nuclei, then examined by fluorescent microscopy. White arrows depict regions used for linescan analysis. (**B**) Linescan analysis, showing the relative intensity (arbitrary units) of FN fluorescence, of the regions depicted in (**A**). (**C**) The bar graph represents the average ± S.E. of the percent FN preserved at the assay front from multiple linescans across three separate donut and scratch migration assays (* = P<0.001).
(TIF)

Figure S3 **The donut migration assay preserves cell viability on the assay periphery.** (**A**) COS-7 cells were plated on FN-coated coverslips and subject to either donut or scratch migration assays. The panels depict phase contrast images of representative fields along the periphery of both assays after staining with trypan blue immediately after donut removal or wounding of the monolayer. (**B**) The data represent the average ± S.E. number of cells that retained the trypan blue stain per 10x field on the assay periphery (* = P<0.001).
(TIF)

Figure S4 **RII, but not RI, PKA subunits are enriched at the leading edge of migrating SKOV-3 cells.** Representative immunofluorescent images of SKOV-3 cells plated on fibronectin (10 µg/ml) coated coverslips for 3 h, then fixed and stained with the antibodies against RI (*red*) and RII (*green*) subunits, and with fluorescent phalloidin to stain filamentous actin (*blue*) are shown. Linescan analysis was used to determine the fluorescence intensity of each target at the leading edge (L.E.) versus the nearby cytoplasm (Cyto) and the average ratio (-/+ std. dev.) of these values for 12 separate linescans was calculated.
(TIF)

Figure S5 **Biosensor activity of pmAKAR3 is PKA-specific.** SKOV-3 cells were transfected with plasmid expressing pmAKAR3 alone or co-transfected with plasmids expressing pmAKAR3 and pmCherry-PKI and, 24 h later, were subjected to the donut invasion assay as described for Figures 5 and 6. FRET ratio images were taken of cells along the perimeter of the monolayer 8 h after the start of the assay. The direction of invasion is indicated by the white arrow.
(TIF)

Text S1 **Text file encoding the DonutQuant macro for ImageJ.**
(TXT)

Movie S1 **PKA is activated at the leading edge of migrating SKOV3 cells.**
(AVI)

Table S1 **Primers used to generate pEGFP-superAKAP** ***is*** **, RIAD, and their scrambled controls.**
(DOC)
