# Supplementary figures and images for: Protein Kinase A Activity and Anchoring Are Required for Ovarian Cancer Cell Migration and Invasion
Source: PLoS One. 2011 Oct 19;6(10):e26552. doi: 10.1371/journal.pone.0026552 (PMC3197526; doi:10.1371/journal.pone.0026552)

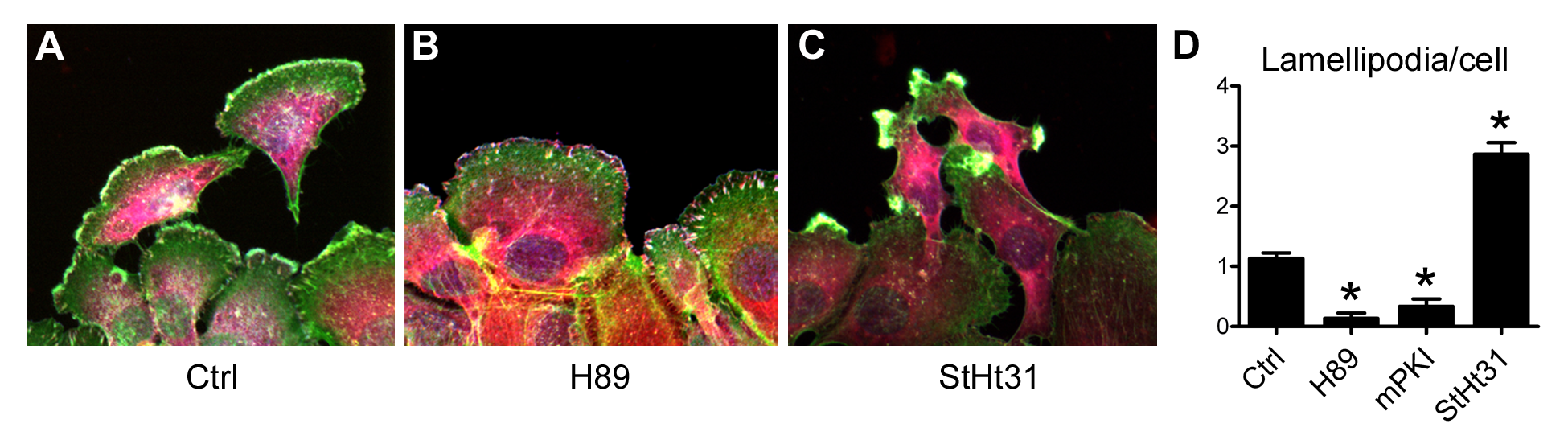

Supplement: Figure S1 — PKA activity and anchoring are required for normal leading edge morphology in migrating SKOV-3 cells. (A-C) Confluent monolayers of SKOV-3 cells plated on FN-coated coverslips were wounded by scratching and re-fed with media containing DMSO (A, Ctrl), 10 µM H89 (B), or 50 µM StHt31 (C). Cells were allowed to migrate into the wound for 4 h before fixation and staining to visualize F-actin (green) and the focal adhesion proteins VASP (red) and vinculin (blue). (D) SKOV-3 cell monolayers were cultured and wounded as described above, then treated with DMSO (Ctrl), H89, StHt31, or 25 µM mPKI. After 4 h, the cells were fixed and stained as above and the number of leading edge lamellipodia in cells at the wound edge was quantified. Values represent the means ± S.E. from at least three experiments (n>100; * = P<0.001). (TIF) [file pone.0026552.s001.tif]

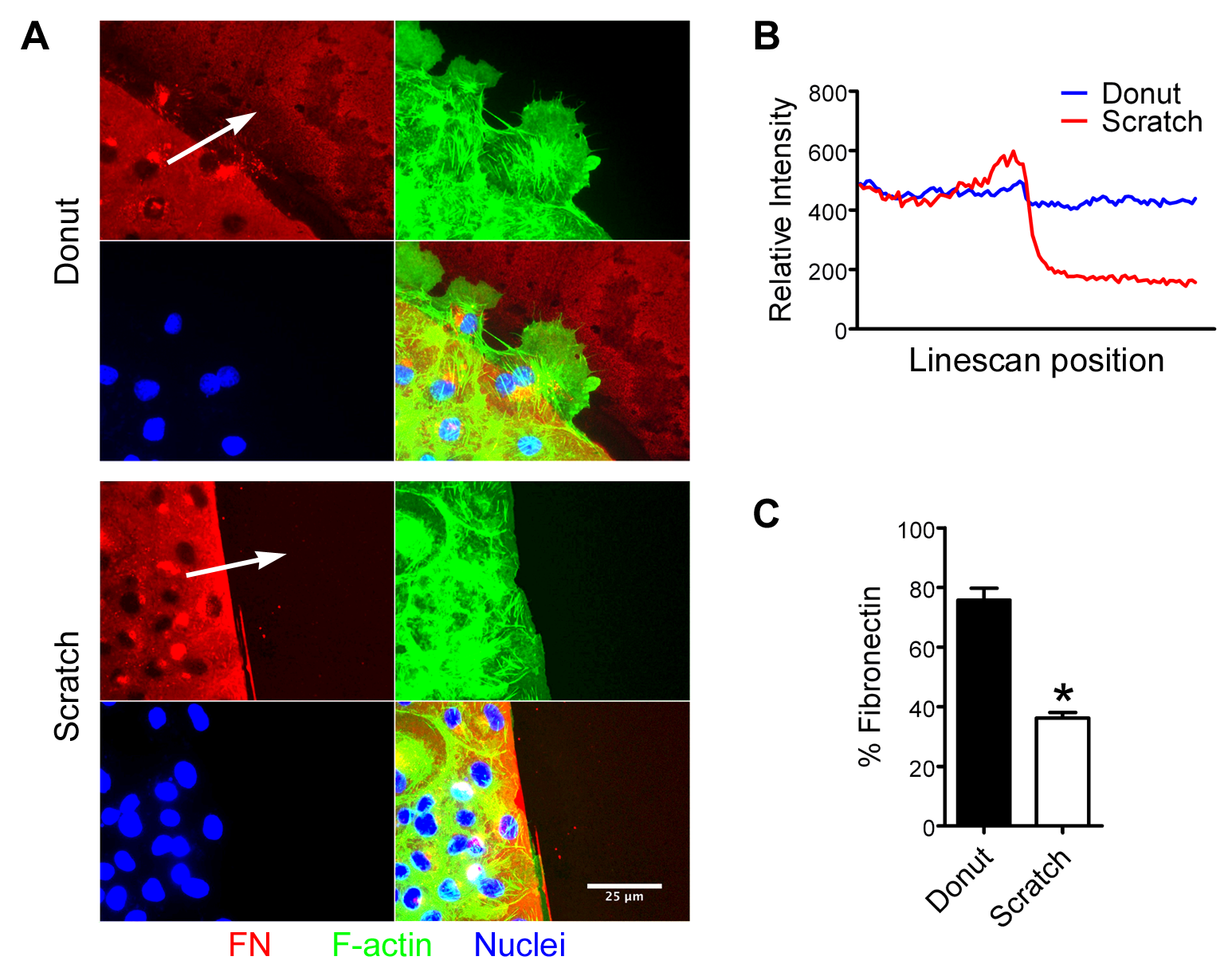

Supplement: Figure S2 — The donut migration assay preserves ECM protein coating better than scratch assays. (A) COS-7 cells were plated on coverslips coated with 20 µg/ml FN and subject to either the donut migration assay or a scratch migration assay for 2 hrs. Coverslips were fixed and processed for immunofluorescence using an antibody against FN, fluorescent phalloidin to stain F-actin, and DAPI to stain nuclei, then examined by fluorescent microscopy. White arrows depict regions used for linescan analysis. (B) Linescan analysis, showing the relative intensity (arbitrary units) of FN fluorescence, of the regions depicted in (A). (C) The bar graph represents the average ± S.E. of the percent FN preserved at the assay front from multiple linescans across three separate donut and scratch migration assays (* = P<0.001). (TIF) [file pone.0026552.s002.tif]

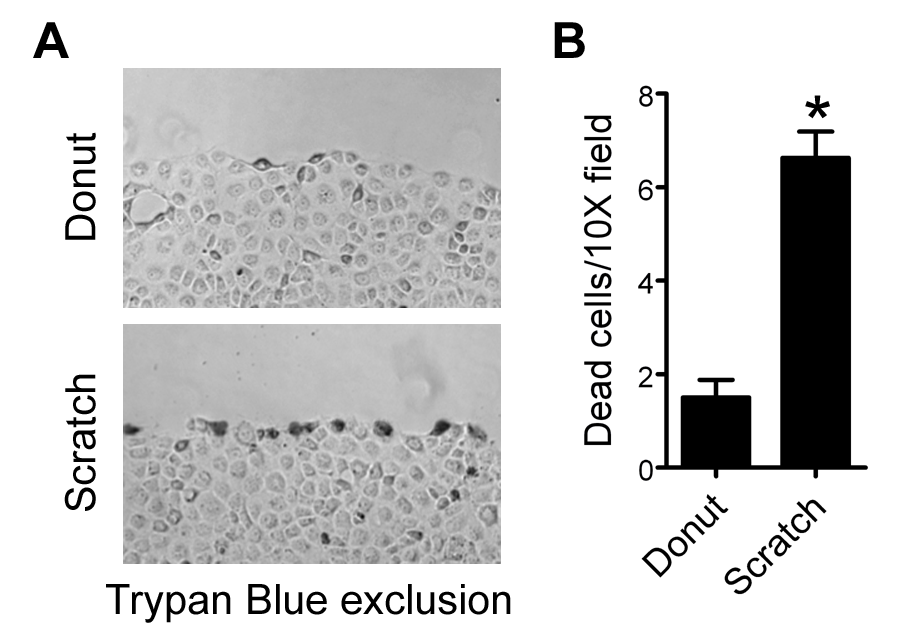

Supplement: Figure S3 — The donut migration assay preserves cell viability on the assay periphery. (A) COS-7 cells were plated on FN-coated coverslips and subject to either donut or scratch migration assays. The panels depict phase contrast images of representative fields along the periphery of both assays after staining with trypan blue immediately after donut removal or wounding of the monolayer. (B) The data represent the average ± S.E. number of cells that retained the trypan blue stain per 10x field on the assay periphery (* = P<0.001). (TIF) [file pone.0026552.s003.tif]

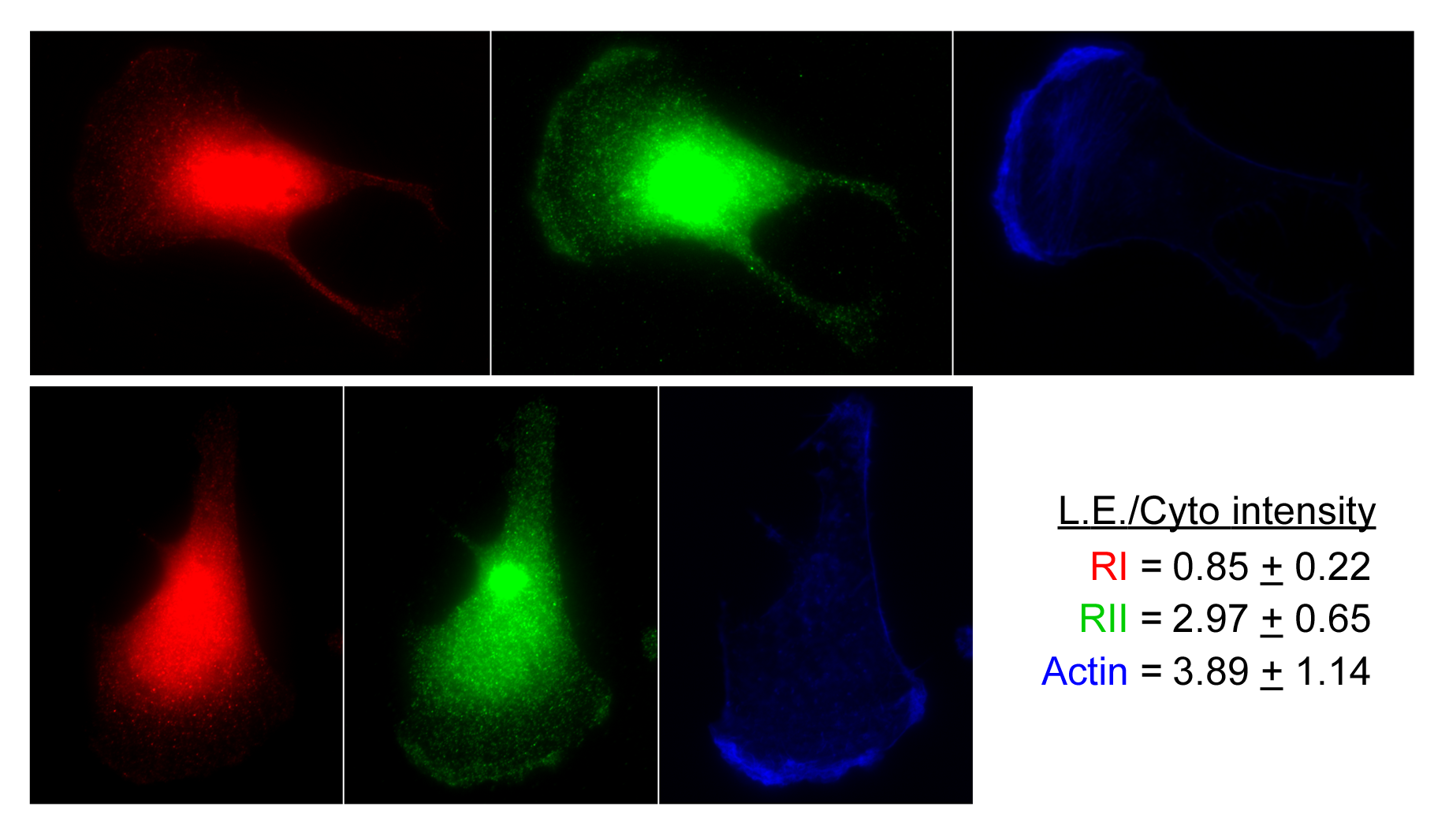

Supplement: Figure S4 — RII, but not RI, PKA subunits are enriched at the leading edge of migrating SKOV-3 cells. Representative immunofluorescent images of SKOV-3 cells plated on fibronectin (10 µg/ml) coated coverslips for 3 h, then fixed and stained with the antibodies against RI (red) and RII (green) subunits, and with fluorescent phalloidin to stain filamentous actin (blue) are shown. Linescan analysis was used to determine the fluorescence intensity of each target at the leading edge (L.E.) versus the nearby cytoplasm (Cyto) and the average ratio (-/+ std. dev.) of these values for 12 separate linescans was calculated. (TIF) [file pone.0026552.s004.tif]

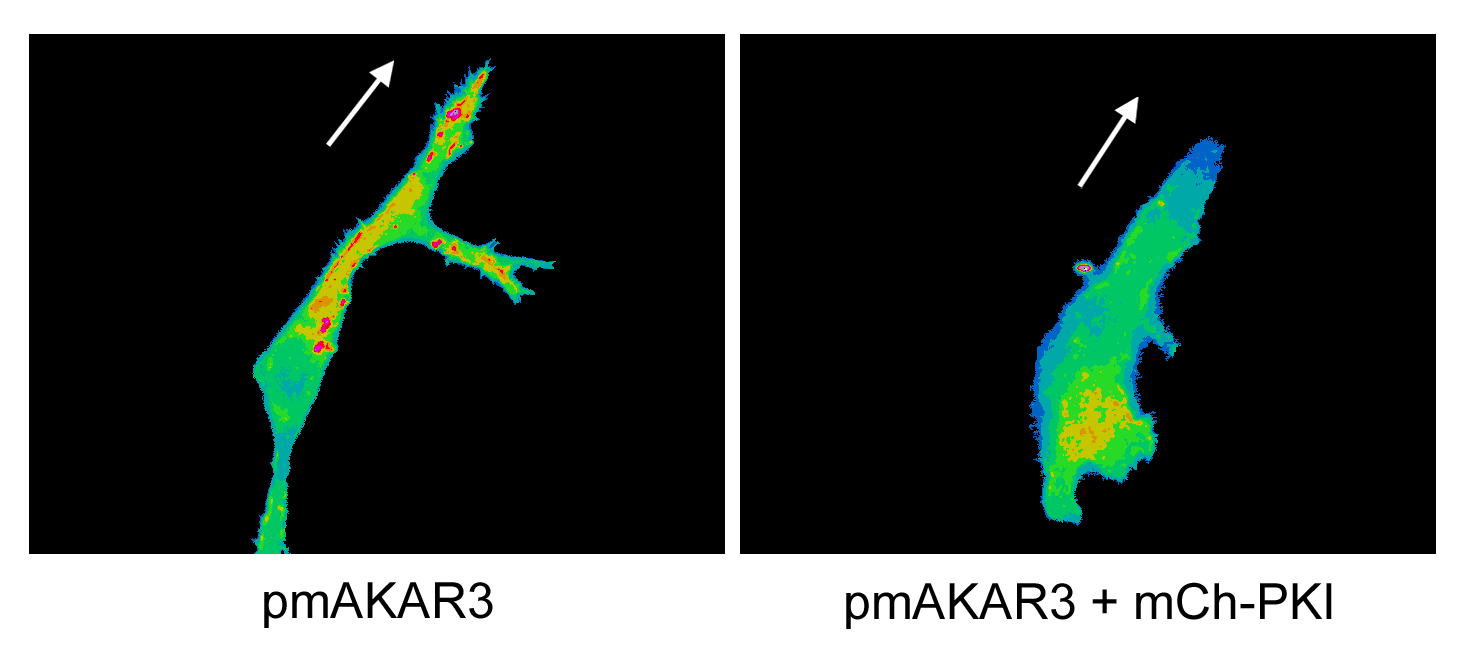

Supplement: Figure S5 — Biosensor activity of pmAKAR3 is PKA-specific. SKOV-3 cells were transfected with plasmid expressing pmAKAR3 alone or co-transfected with plasmids expressing pmAKAR3 and pmCherry-PKI and, 24 h later, were subjected to the donut invasion assay as described for Figures 5 and 6. FRET ratio images were taken of cells along the perimeter of the monolayer 8 h after the start of the assay. The direction of invasion is indicated by the white arrow. (TIF) [file pone.0026552.s005.tif]
