# Supplementary material for: Protein Kinase A Activity and Anchoring Are Required for Ovarian Cancer Cell Migration and Invasion
Source: PLoS One. 2011 Oct 19;6(10):e26552. doi: 10.1371/journal.pone.0026552 (PMC3197526; doi:10.1371/journal.pone.0026552)
Supplement: Table S1 — Primers used to generate pEGFP-superAKAP is , RIAD, and their scrambled controls. (DOC) [file pone.0026552.s008.doc]

**Table S1: Oligonucleotide sequences for cloning RIAD, sA*is*, or their scrambled sequences into pEGFP-N1.**

| **Primer** | **Sequence (5’ – 3’)** |
| --- | --- |
| RIAD fwd | TCGACCTAGAACAATACGCAAACCAACTAGCAGACCAAATAATAAAAGAAGCAACAGAATAAG |
| RIAD rev | GATCCTTATTCTGTTGCTTCTTTTATTATTTGGTCTGCTAGTTGGTTTGCGTATTGTTCTAGG |
| RIAD_scr fwd | TCGACCTAGCACAAGAATACAACCTACAAGCAGACATACAAATAAAAGCAGAAACAGAATAAG |
| RIAD_scr rev | GATCCTTATTCTGTTGCTTCTTTTATTATTTGGTCTGCTAGTTGGTTTGCGTATTGTTCTAGG |
| sA*is* fwd | TCGACCAAATAGAATACGTAGCAAAACAAATAGTAGACTACGCAATACACCAAGCATAAG |
| sA*is* rev | GATCCTTATGCTTGGTGTATTGCGTAGTCTACTATTTGTTTTGCTACGTATTCTATTTGG |
| sA*is*_scr fwd | TCGACCAAGACGTAGAAATACACGTAAAAGCAGCATACTACCAACAAATAGCAATATAAG |
| sA*is*_scr rev | GATCCTTATATTGCTATTTGTTGGTAGTATGCTGCTTTTACGTGTATTTCTACGTCTTGG |
